# Hematopoietic and Mesenchymal Stem Cells in Biomedical and Clinical Applications

**DOI:** 10.1155/2016/3157365

**Published:** 2016-11-24

**Authors:** Albert A. Rizvanov, Jenny Persson, Fikrettin Şahin, Saverio Bellusci, Paulo J. Oliveira

**Affiliations:** ^1^Institute of Fundamental Medicine and Biology, Kazan Federal University, Kazan, Russia; ^2^Division of Experimental Cancer Research, Department of Laboratory Medicine, Lund University, Malmö, Sweden; ^3^Department of Genetics and Bioengineering, T.C. Yeditepe Üniversitesi, Istanbul, Turkey; ^4^Excellence Cluster Cardio-Pulmonary System, Justus Liebig University, Giessen, Germany; ^5^CNC-Center for Neuroscience and Cell Biology, UC Biotech Building, University of Coimbra, Cantanhede, Portugal

Stem cell-based therapy is emerging as a major area of investigation, not only to understand the basic signaling mechanisms that regulate cell fate but also to involve them as a novel approach for the treatment of multiple human diseases. The interest in stem cells is growing each year. Stem cell research is transversal to many fields of research ranging from embryology to reproduction to cell division to cell differentiation to therapies, and even to plant biology. It is thus not surprising that the stem cell field is gaining more and more relevance and maturity, despite some major disappointments [1].

Despite advances in biomedical and clinical research, many diseases and conditions have no known cure or still do not have adequate therapies which would result in sufficient recovery and high quality of life for patients. Boosting the natural regenerative abilities of the human body seems like a promising therapeutic strategy. Stem cells possess an ability for self-renewal and differentiation into several cell types thus playing an important role in natural replacement of aged or apoptotic cells as well as regeneration of damaged tissues. Mesenchymal and hematopoietic stem cells play an important role in many regeneration processes in the human body. Moreover, according to recent studies, mesenchymal and hematopoietic stem cells form a unique bone marrow niche [2]. Not surprisingly, hematopoietic and mesenchymal stem cells are considered to be the most promising adult stem cell types for developing cell- or gene-based therapies.

The present special issue deals with the biomedical and clinical applications of hematopoietic and mesenchymal stem cells, serving as a pit stop for many stem cell researchers, not only to review the latest literature but to improve their current knowledge on mesenchymal (MSCs) and hematopoietic stem cells (HSCs).

Mesenchymal stem cells are a promising* source of stem cells for regenerative/healing therapy* as illustrated by several interesting papers present in the current edition. MSCs are distinct from the hematopoietic stem cells and have the capacity to differentiate into multiple cell types including adipocytes, chondrocytes, myocytes, and osteoblasts. MSCs do not give rise to differentiated cells in the hematopoietic lineage.

*The study by H. Zhao et al.* reports the therapeutic effects of MSC on Severe Acute Pancreatitis (SAP) in rats. The authors elegantly show that MSC transplantation improves the prognosis of SAP. Engrafted MSCs have the capacity of homing, migrating, and establishment during the treatment of SAP.

*The study by C. Yin et al.* investigated the role of umbilical cord-derived mesenchymal stem cells (UC-MSCs) in regulating angiogenesis and relieving hindlimb ischemia. They successfully demonstrated that the isolated UC-MSCs notably contributed to restoring blood supply and alleviating the symptoms of limb ischemia through enhancing angiogenesis, opening the way for a novel approach to treat that condition.

*The review by A. J. Peired et al.* summarizes the clinical evidence for the use of exogenous MSCs to prevent renal injury and promote renal recovery. Ongoing clinical trials are expected to provide further insight into safety, feasibility, and efficacy of MSC-based therapy in renal pathologies and allow the design of consensus protocol for clinical purpose.

*The review by S. Li et al.* summarizes the current status, therapeutic potential, and the detailed factors of MSCs-based therapeutics for ischemic cerebrovascular disease, ischemic cardiomyopathy, and diabetic foot. Diabetes and its related complications are in fact a large cause of morbidity and mortality nowadays in the Western countries, mostly derived from incorrect lifestyles.

*The review by Q. Wu et al.* explores further the roles and functional mechanisms of MSCs in diabetic foot disease. Insights into current and future studies are presented.

Related to the previous topic,* the review by K. Matsushita* discusses the current understanding of the relationship between MSCs and metabolic syndrome (obesity, dyslipidemia, and diabetes). The author also discusses their potential implications in disease management presenting also the pitfalls associated with their use.

Each organ has potentially a source of mesenchymal stem cell, called resident mesenchymal stem cells. Placenta, cord blood, and amniotic fluid at birth as well as adipose tissue or the dental pulp of baby teeth postnatally have been shown to be a practical, easily accessible,* source of MSCs*.

*The study from H. Ren et al.* compares the biological characteristics, including morphology, proliferation, antiapoptosis, multilineage differentiation capacity, and immunophenotype of umbilical cord, dental pulp, and menstrual blood-MSCs in order to provide a theoretical basis for the clinical selection and application of these cells. Their results suggest that dental pulp-MSCs, collected in an easy and noninvasive way, may be a desired source for clinical applications of cell therapy.

Another emerging area of research is the manipulation of MSCs* in vitro* before* in vivo* transplantation to enhance their regenerative/healing capabilities. Three studies in the present special edition deal with this important research question

*The study by P. Müller et al.* focuses on CD133+ stem cells, which are very promising for regenerative medicine. Using magnetic polyplexes carrying microRNA, they specifically targeted human CD133+ stem cells without altering their stem cell potential. The approach described in this work may be used for a better selection of cells in order to enhance their therapeutic effects.

*Another study by L. Jinfeng et al.* is based on the premises that appropriate gene transduction before transplantation of MSCs derived from the human umbilical cord is a promising procedure for cell therapy. Fibroblast Growth Factor-20 (FGF-20) has been reported to protect dopaminergic neurons against a range of toxic insults* in vitro*. In this paper, MSCs were gene transduced with FGF-20 and transplanted into a mice model of Parkinson's disease. The authors reported that transduced MSCs have the potential for improving Parkinson's disease, closely related to the degradation of NF-*κ*B, a transcription factor that controls genes encoding proinflammatory cytokines, highly expressed in the nigrostriatal dopaminergic regions in Parkinson's disease patients.

*The study by D. Ye et al.* investigated whether the DNA methyltransferase inhibitor (DNMTi) 5-aza-2′-deoxycytidine and the histone deacetylase inhibitor (HDACi) trichostatin A regulated hepatic differentiation of rat bone marrow-derived mesenchymal stem cells (rBM-MSCs) as well as their therapeutic effect on liver damage. The authors showed that HDACis enhanced hepatic differentiation in a time-dependent manner, while DNMTis did not induce the hepatic differentiation of rBM-MSCs* in vitro*.

*How endogenous MSCs can be recruited to the site of injury is another key aspect in MSC research, with very important implications in tissue regeneration*.

*The study by E. Muinos-Lopez et al.* evaluated the effect of intra-articular or a combination of intra-articular and intraosseous infiltration of Platelet-Rich Plasma (PRP) on the cellular content of synovial fluid (SF) of osteoarthritic patients. The authors demonstrated that the synovial fluid of osteoarthritic patients contains a population of MSCs that can be modulated by Platelet-Rich Plasma infiltration of the subchondral bone compartment.

MSCs cells can either act as a source of differentiated cells or/and* modulate the local inflammatory status via the secretion of cytokines, possibly highlighting their potential therapeutic role in immune diseases*. MSCs have been used so far in clinical trials for osteoarthritis, autoimmune disease (Crohn's disease, multipole sclerosis, systemic lupus erythematosus, and systemic sclerosis), or systemic diseases such as graft-versus-host diseases and sepsis.

*The study by M. A. Al Jumah et al.* characterized the role of mesenchymal stem/multipotent stromal cells from decidua basalis of human term placenta-derived mesenchymal stem cells (DBMSCs). They showed that DBMSCs differentiate into three mesenchymal lineages (adipocytes, osteocytes, and chondrocytes). DBMSCs express and secrete a distinct combination of cytokines, growth factors, and immune molecules that reflect their unique microenvironment. The authors suggested that DBMSCs could be attractive, alternative candidates for MSC-based therapies that treat diseases associated with inflammation and oxidative stress

*The review by B. Sangiorgi and R. A. Panepucci* is focused on the immunomodulatory properties of mesenchymal stromal cells (MSCs) in regard to their potential use in the treatment of graft-versus-host disease. In their review, the authors illustrate how Pathogen-associated molecular patterns modulate the immunosuppressive phenotype of human MSCs by signaling through toll-like receptors.

Hematopoietic stem cells are among the first stem cell types used in the clinic, mainly to treat blood disorders and restore hematopoietic function after radiation and chemotherapy. Besides their natural ability to differentiate into multiple hematopoietic lineages, these cells are also considered for therapy of many other diseases, such as neurodegenerative diseases, autoimmune diseases, and different types of traumatic injuries. Finally, some types of cancer stem cells are thought to originate from hematopoietic stem cells. It is therefore crucial to understand the molecular and cellular bases controlling the survival, proliferation, and differentiation of these cells in order to develop new therapeutic strategies for oncology.

*The study by Y.-C. Liu et al.* is focused on investigating the prognostic factors that may predict patient outcome after the allogeneic hematopoietic stem cell transplantation (HSCT). It is important to identify new prognostic factors that may predict graft-versus-host disease and relapse-free survivals.

*The study by J. Li et al.* showed that the intraperitoneally transplanted multiplacentas pooled cells can survive and engraft into the host body through blood circulation. The aplastic anemia model mice which received the multiplacentas pooled cells display increased peripheral blood hemoglobin level. In turn, increased hemoglobin is thought to be causative for the observed increased life span of aplastic anemia model mice after intraperitoneal transplantation of these cells.

*The review by P. Sontakke et al.* overviewed the mouse xenograft models that have been used for studying the molecular pathology of Chronic Myeloid Leukemia (CML). The authors also described the advantages of using the mouse models of CML to develop and improve therapeutic approaches for this disease. Interestingly, the authors proposed that the stem cell technologies, which are applied in the establishment of animal models, can maintain patient tumor-derived CML-subclones that are genetically identical to those in patients.

*Albert A. Rizvanov* *Albert A. Rizvanov* *Jenny Persson* *Jenny Persson* *Fikrettin Şahin* *Fikrettin Şahin* *Saverio Bellusci* *Saverio Bellusci* *Paulo J. Oliveira* *Paulo J. Oliveira*
